# Developing competencies in public health: a scoping review of the literature on developing competency frameworks and student and workforce development

**DOI:** 10.3389/fpubh.2024.1332412

**Published:** 2024-03-04

**Authors:** Melissa MacKay, Caitlin Ford, Lauren E. Grant, Andrew Papadopoulos, Jennifer E. McWhirter

**Affiliations:** Department of Population Medicine, Ontario Veterinary College, University of Guelph, Guelph, ON, Canada

**Keywords:** public health, competencies, competency frameworks, professional development, competency-based education, pedagogy, training

## Abstract

Effective and precise public health practice relies on a skilled and interdisciplinary workforce equipped with integrated knowledge, values, skills, and behaviors as defined by competency frameworks. Competency frameworks inform academic and professional development training, support performance evaluation, and identify professional development needs. The aim of this research was to systematically identify and examine trends in the extent, nature, and range of the literature related to developing competencies in public health. This includes developing public health competency frameworks, and how competencies are developed and maintained in students and practitioners. We used a scoping review methodology to systematically identify and report on trends in the literature. Two independent reviewers conducted title and abstract and full-text screening to assess the literature for relevance. Articles were included if they were original primary research or gray literature and published in English. No date or geographic restrictions were applied. Articles were included if they focused on developing competency statements or frameworks for public health and/or training public health students or practitioners to develop competencies. The review encompassed a range of methods and target populations, with an emphasis on building competencies through student and professional development. Foundational competency development was a primary focus, and we found a gap in discipline-specific competency research, especially within developing discipline-specific competency statements and frameworks. Several evidence-based practices for competency development were highlighted, including the importance of governance and resources to oversee competency framework development and implementation, and workforce planning. Experiential learning and competency-based training were commonly identified as best practices for building competencies. A comprehensive understanding of public health competency development—through developing and incorporating foundational and discipline-specific competencies, mapping student and practitioner training to competency frameworks, and incorporating best practices—will enable public health to create skills and an adaptable workforce capable of addressing complex public health issues.

## 1 Introduction

Health professionals require a specific set of skills, values, and knowledge–commonly referred to as core competencies–to be highly effective in their respective sectors. Core competencies represent a baseline standard of the knowledge, skills, and attitudes required among professionals in the field (1). Competency statements and frameworks are generated by allied health organizations and regulatory bodies globally, including core competencies for incoming medical students (2), entry-to-practice competencies for registered nurses (3, 4), core competencies for clinical pharmacists (5), and professional competencies for veterinarians (6, 7). While they vary across professions, these competencies encompass a set of agreed-upon interpersonal, clinical, scientific, and logical reasoning skills, values, and knowledge that practitioners should exhibit.

Specific to public health, competency statements and frameworks exist across public health organizations and institutions internationally. Governing bodies in Canada (1), the United States (8), the United Kingdom (9), the European Union (10), and Australia (11) have all released competency frameworks to guide public health workforce planning. Although each framework differs in its content and context, they all aim to strengthen the public health sector’s ability to prepare for and respond to public health challenges-- today and in the future (12).

Core competencies provide standards for training the public health workforce (1, 8, 12). These frameworks can inform academic and professional development curricula (1), assess workforce performance (13), and identify professional development needs. Core competencies can also help public health organizations maintain consistency, identify program needs, and help facilitate interdisciplinary work (1, 8). Competency statements and frameworks can ensure public health practitioners have a shared understanding of their roles and responsibilities and that they are working toward a common goal of contributing to a healthier population (1).

Competency frameworks can become outdated as workforce needs evolve in response to changing population health demands. As such, there have been global calls (14–16) to strengthen and transform the public health workforce to adapt to evolving public health challenges like the COVID-19 pandemic (17), climate change (18), and economic inflation (17). In Canada, the Canadian Public Health Association (CPHA) explicitly called on the government to refresh their public health core competency framework to encompass these challenges (14). Further, Canada’s Chief Public Health Officer called for modernized and improved core competencies and curriculum in graduate public health education, and transformation of the public health workforce to better address complex public health challenges such as climate change and the COVID-19 pandemic (16, 18).

Competency-based education and training guide curriculum planning, accreditation, and performance evaluation to enhance outcomes of public health practice (19, 20). Further, competency frameworks bridge the gap between evidence and practice and allow for interdisciplinary skill set development (12, 21). Flexible learning, mentorship, and feedback are key factors in effective competency-based education in healthcare (20). Experiential learning, practice-based learning, and reflective practices are also effective pedagogical practices for developing competencies in public health students (22). In Canada, graduate-level training matched to public health competencies is especially important given the lack of a national, comprehensive competency training program (23).

As Canada and other countries around the world begin to transform their public health workforce, agencies must draw on evidence-based practices for building competencies. There are currently gaps in understanding best practices for developing, implementing, and evaluating public health competency frameworks, and there are no published reviews that capture the breadth of this process. Related research has described the process for developing public health competency frameworks including the need for transparent reporting, validated instruments, and consensus-building approaches (24), but no such reviews exist examining the full scope of research on building competencies in public health. Thus, there is a need for synthesized results to inform evidence-based practices related to developing public health competencies going forward.

The aim of this research is to systematically identify and examine trends in the extent and nature of the literature related to developing competencies in public health. The scope includes developing public health competency frameworks, and identifying how competencies are developed and maintained in students and practitioners. The objectives of this research are:

1. To conduct a scoping review of the relevant literature regarding developing competencies in public health; and,
2. To report on trends in the extent and nature of the literature identified.

## 2 Materials and methods

This scoping review followed the framework by Arskey and O’Malley (25), and updated by Levac et al. (26) to examine the extent, nature, and range of research related to developing public health competencies. Initially, the review intended to focus on health communication competencies; however, the literature was limited in this area, so the review reflects the full range of developing public health competencies. This research adheres to the PRISMA for Scoping Reviews reporting guidelines (27).

### 2.1 Search strategy

A comprehensive search strategy was developed by the research team in collaboration with a Specialist Librarian from the University of Guelph. The search contained three concepts: health communication, public health, and pedagogy/education/competencies (Table 1). The search was piloted in Ovid via MEDLINE, as well as by screening the reference lists of relevant articles. Six databases were searched including Ovid via MEDLINE, PsycINFO, Web of Science, Communication and Mass Media Complete, ERIC, and CAB Direct to identify relevant literature. The search was conducted on November 24, 2022. The search was verified by hand searching the following journals, which were most often cited in the database search results: *Journal of Public Health Management & Practice, American Journal of Public Health, Journal of Health Communication, Health Promotion Practice*, and *Health Communication*.

**Table 1:** Search concepts, controlled vocabulary, and keywords.

Concept	MeSH Term(s)	Keywords
Health communication	Health Communication	“Health communication” OR communication OR “health information” OR “health informatics” OR dissemination OR “knowledge translation” OR “behavior change” OR awareness OR attitude* OR knowledge OR “social marketing” OR “social media” OR “communication channel*” OR “health literacy” OR “mass media” OR “cultural competency” OR “online health communication” OR “risk perception” OR “risk communication” OR “crisis communication” or “health ethics”
Public health	Public Health Practice	“Public health” OR “public health practice” OR “public health practitioner*” OR “public health workforce” OR “public health capacity” OR “public health capabilit*” OR “evidence-based public health practice” OR “public health impact” OR “practitioner knowledge”
Pedagogy/Education/Competencies	Students, Public Health Education, Public Health Professional Professional education Curriculum	Pedagogy OR education OR learning OR teaching OR curriculum OR curricula OR “transmission of knowledge” OR “professional development” OR “Master of Public Health” OR “public health training” OR “public health education” OR “training program*” OR “public health graduate training” OR “public health graduate education” OR “competency-based curricul*” OR “competency-based assessment*” or competency or understanding or qualification* or categor* or “competency-based curricul*” or “competency-based assessment?” or skill* or “know how” or “competency adj5 belief*” or “competency adj5 value*” or “competency adj5 attitude*”

Using appropriate combinations of concepts and keywords, Google was also searched to identify relevant gray literature. The first 10 pages were searched for each of the following keyword/phrase combinations: “public health” AND core competencies, core competencies AND “public health workforce,” core competencies AND “public health students,” and teaching and learning AND “public health.”

All citations obtained in the database and gray literature search were downloaded to Mendeley (28), where the deduplication tool was used. The deduplicated references were then uploaded into DistillerSR (29) to manage the screening and data extraction processes.

### 2.2 Study inclusion criteria

To be included, literature had to be published in English from any location, and focused on competency development in public health practitioners and/or students Literature also had to focus on developing competency statements or frameworks for public health and/or training public health practitioners or students to develop competencies. No date restrictions were used, and original primary research and gray literature were included.

Studies were excluded if they focused on competency development in other disciplines (e.g., nursing, medicine, dentistry, etc.) unless it was public health competency-focused (e.g., public health nursing). Research on public health interventions aimed at individuals, communities, groups, etc. that did not feed into knowledge about effective training or education of students and the workforce were also excluded. Finally, descriptive studies about a public health program, training opportunity, or workshop without some basis in program development theory or frameworks were excluded.

### 2.3 Study selection

Two researchers conducted a pilot test before screening began. Articles were screened in two stages by two independent reviewers. First, the title and abstracts of each article were screened for relevance to the inclusion criteria using a structured form. Kappa was 0.81 for this screening stage, indicating high agreement (30). All conflicts were resolved through discussion.

Next, the full text of articles found to be potentially relevant to this review were independently screened by the same reviewers. Several steps were taken to obtain the full text of all articles if they were not available through traditional means including searching the University of Guelph Library, using Google and Google Scholar, and contacting the researcher directly via ResearchGate and/or their institutional email if available. Articles were screened using a structured form that contained the following criteria: literature type, language, population, measurement, evaluation, or detailed report of competency statement or framework development in public health students and/or the workforce. Kappa was 0.80 for this stage of screening, indicating high agreement (30), and all conflicts were resolved through discussion.

### 2.4 Data extraction and analysis

This review focused on mapping prominent trends in developing competencies in public health, including competency frameworks and competency development in students and practitioners. The data extraction form was developed by the lead researcher, piloted by the two independent reviewers, and finalized by the research team prior to data extraction. The following fields were extracted using a structured form in DistillerSR: title, author(s), year, article type, country/region of origin, study design, study aim, methods, theories or frameworks included, pedagogy identified, institutions involved in the research, institutions involved in the professional or student training, existing competency frameworks included, focus of competency development (e.g., developing a competency framework, workforce development, student development), focus of competency framework (e.g., general or discipline-specific), target population (e.g., graduate student, general public health practitioner), focus of practitioner or student training, focus on public health communication, recommendations for improved competency development, bias identified, and future research directions.

The dataset was split in half so that the two reviewers were each the primary extractors for half and verified the other half. The recommendations for improved competency development were captured deductively based on a related review (24), and inductively to capture approaches for developing competencies in public health practitioners and students found in the included literature. The inductively captured approaches were analyzed by uploading the open text responses into NVivo Plus 12 (31) and conducting an automatic thematic analysis. Codes with multiple references were explored by the lead researcher to generate overall promising approaches for developing competencies and were verified by a second researcher and the research team.

Descriptive statistics demonstrate the trends in the collected information relevant to the review aim. Microsoft Excel (Microsoft, Redmond, USA) was used to analyze and visualize the data.

## 3 Results

**Figure 1:**
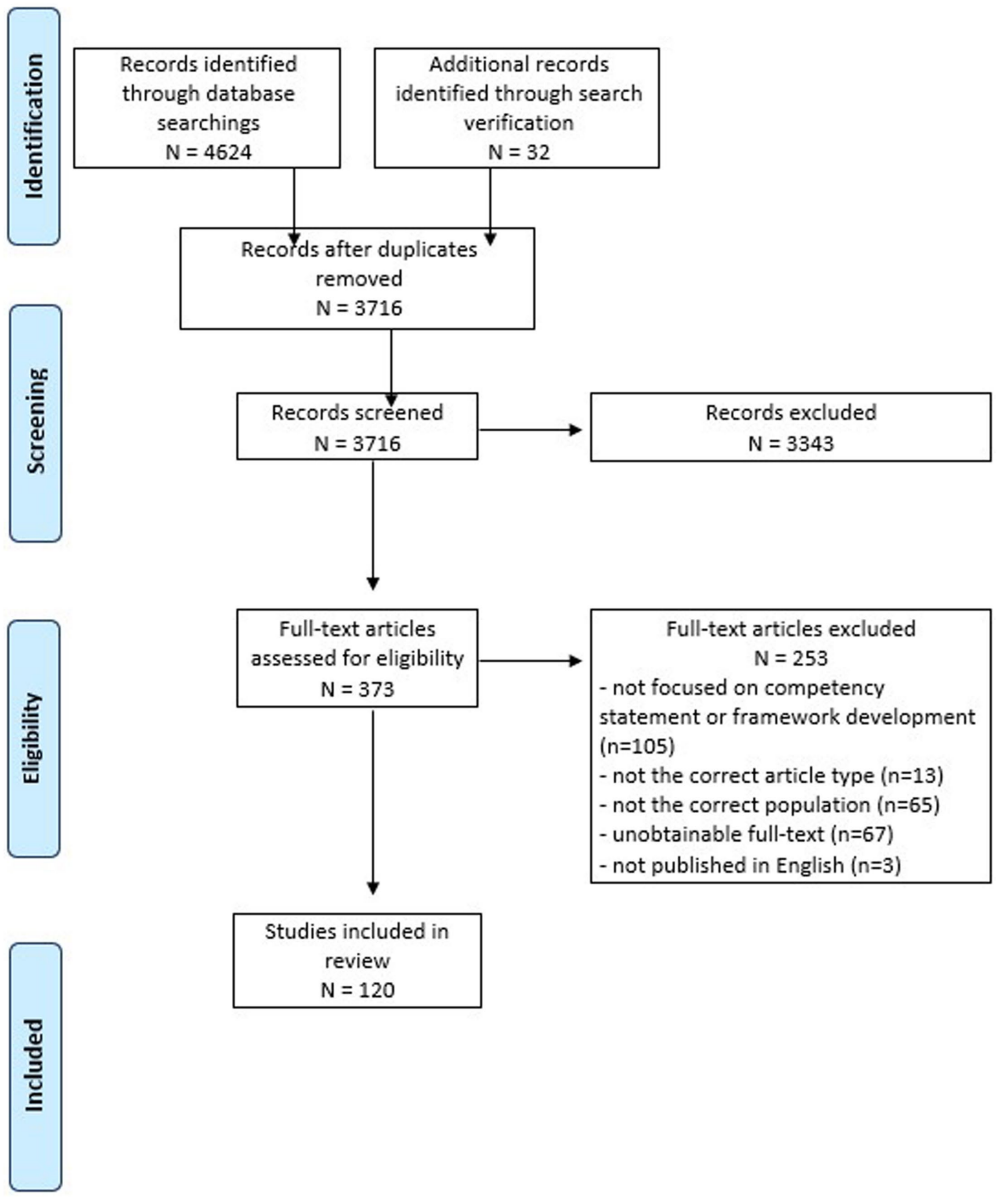
PRISMA flow diagram of scoping review process.

The search identified 3,716 articles after deduplication and a total of 120 articles met the inclusion criteria and underwent data extraction and analysis (Figure 1). Generally, articles either described the development of competency statements and frameworks or the development of competencies through training and professional development.

Most included articles used mixed method designs (44%) and were conducted in the United States of America (64%) (Table 2). Supplementary Table S1 provides the authors, year, article title, and study details of all the included literature (*n* = 120). General public health practitioners (48%), followed by Master of Public Health (MPH) students (22%), were the common target populations for the included articles. Other public health practitioners commonly cited (12%) include public health leaders, environmental public health practitioners, public health nurses, public health nutritionists, and Indigenous health workers. Universities (89%) conducted most of the included research, followed by collaborations between the cited institutions and organizations (e.g., universities and governments) (34%). Other organizations (9%), including research funders and networks, government-funded and/or operated training centers, and consultants also conducted the research. Existing competency frameworks were often not included (45%) in the articles, but when they were, the framework by the US Core Competencies for Public Health Professionals (35%) was the most frequently incorporated. Finally, bias was not identified (44%) in some studies; however, other studies cited participant bias (27%).

**Table 2:** Article attributes summary.

Article attributes	*n* (%)^+^ 120 (100%)
Methods	
Mixed methods	53 (44%)
Qualitative	39 (33%)
Quantitative	25 (21%)
Gray literature	3 (3%)
Country/Region of Origin*	
United States of America	77 (64%)
Canada	11 (9%)
Australia	11 (9%)
United Kingdom	10 (8%)
South Africa	2 (2%)
England	1 (1%)
Brazil	1 (1%)
Central America	1 (1%)
Haiti	1 (1%)
China	1 (1%)
Tanzania	1 (1%)
Netherlands	1 (1%)
Africa	1 (1%)
India	1 (1%)
Ireland	1 (1%)
Global	1 (1%)
Target population*	
General public health professional	58 (48%)
Master of public health student	27 (22%)
Undergraduate student	21 (18%)
Graduate student (not MPH)	15 (13%)
Other	14 (12%)
Nurse	5 (4%)
Doctor	4 (3%)
Indigenous focused public health professional	3 (3%)
Epidemiologist	2 (2%)
Nutrition professionals	2 (2%)
Public health educators	2 (2%)
Healthcare students	1 (1%)
Existing competency framework included*	
Not applicable	54 (45%)
Core competencies for public health professionals (U.S.A.)	42 (35%)
Other	16 (13%)
PHAC core competencies (Canada)	9 (8%)
ASPHER’s European list of core competencies for the public health professional	7 (6%)
Multiple frameworks	4 (3%)
Generic competencies for public health in Aotearoa-New Zealand	1 (1%)
Article attributes	*n* (%)^+^ 120 (100%)
Organization/Institution(s) Conducting research*	
University	107 (89%)
Collaborations between organizations	41 (34%)
Government	28 (23%)
Community-based organization	16 (13%)
Other	11 (9%)
Public health unit/agency	10 (8%)
Healthcare organization	5 (4%)
Models and frameworks cited by more than one study*	
None used	66 (55%)
Other (e.g., Delphi Technique, Blooms Taxonomy)	47 (54%)
Kirkpatrick training evaluation model	7 (6%)
Bias identified*	
None	53 (44%)
Selection bias	32 (27%)
Generalizability	31 (26%)
Response bias	22 (18%)
Lost to follow up	13 (11%)
Information bias	11 (9%)
Data collection	7 (6%)
Not applicable (Gray Literature)	5 (4%)

*Multiple categories possible for each article, thus may not total 100%. ^+^Percentages rounded to the nearest whole number.

Table 3 describes the range of competency development work in public health from building competency statements and frameworks to student education and professional training. Professional development (49%) was often the focus of the included literature, followed by developing competencies in public health students (36%). Of the studies that assessed competencies, general surveys (55%) and pre- and post-surveys (33%) were employed as a step within building competency frameworks, as well as to assess self-rated competency changes after student and professional training. Universities (76%) were the institutions that most commonly conducted training in the included articles; however, collaborations (28%) between cited institutions and organizations were also common. Other organizations (9%) who participated in developing and conducting training included federally funded organizations and consultants. Of the studies that included professional development/training opportunities, some are conducted online (39%) and focused on a variety of other topics (45%) including cultural competency, health equity and literacy, and epidemiology. When student training was the focus, it was often centered within an academic course (41%) and incorporated experiential learning (40%). Articles that focused on development of competency statements and frameworks are limited (10%), but when present are focused on foundational competencies, epidemiology, leadership, and other discipline-specific areas. One article (1%) focused on refreshing an existing competency framework.

**Table 3:** Range of competency development work depicted in literature.

Competency development focus	*n* (%) 120 (100%)
Aspect of competency focus* (*n* = 120/120, 100%)	***n*/120 (100%)**
Purpose of study in terms of competency development	
Professional development	59 (49%)
Student development	43 (36%)
Building competency statements/frameworks	12 (10%)
Curriculum development	12 (10%)
Other (e.g., Assessing competencies in practitioners, evaluating frameworks)	8 (7%)
Refreshing existing frameworks	1 (1%)
Competency assessment method* (*n* = 111/120, 93%)	***n*/111 (100%)**
How competencies statements/frameworks were developed or assessed	
Survey	61 (55%)
Pre- and Post-Survey	37 (33%)
Interviews	29 (26%)
Other (e.g., Critical Reflection, Course Evaluation)	24 (22%)
Focus groups	9 (8%)
Organization/Institution conducting training* (*n* = 120/120, 100%)	***n*/120 (100%)**
Organizer of the professional development or student training	
University	91 (76%)
Collaboration	34 (28%)
Government	26 (22%)
Community-based organization	13 (11%)
Public health unit	11 (9%)
Other (e.g., National Research Network, Training Center)	11 (9%)
Not-For-Profit	4 (3%)
Healthcare organization	2 (2%)
Focus of professional development/student training* (*n* = 97/120, 81%)	**n/97 (100%)**
Focus of training for practitioners and students	
Other (e.g., participatory research for health equity, cultural competency)	44 (45%)
General public health	27 (28%)
Evidence-informed decision-making	13 (13%)
Crisis and/or risk communication	13 (13%)
Cultural competency	6 (6%)
Focus of competency framework* (*n* = 23/120, 19%)	***n*/23 (100%)**
Discipline related to competency statement and framework development	
Foundational	8 (35%)
Other	8 (35%)
Epidemiology	3 (13%)
Evidence-informed decision-making	1 (4%)
Health communication	1 (4%)
Public health medicine	1 (4%)
Competency development focus	*n* (%) 120 (100%)
Public health nutrition	1 (4%)
Type of professional development* (*n* = 66/120, 55%)	***n*/66 (100%)**
Method and/or mode of professional development	
Online	26 (39%)
Organizational	21 (32%)
Community-based partnership	13 (20%)
Academic course	9 (14%)
Academic program	2 (3%)
Type of student training* (*n* = 49/120, 41%)	***n*/49 (100%)**
Method and/or mode of student training	
Academic course	20 (41%)
Academic program	14 (29%)
Internship/practicum	11 (22%)
Online course	5 (10%)
Community-based	3 (6%)
Pedagogy identified* (*n* = 50/120, 42%)	***n*/50 (100%)**
Method and practice of teaching and learning identified	
Experiential learning	20 (40%)
Adult learning	17 (34%)
Other	17 (34%)
Community-engaged scholarship	7 (14%)
Case-based learning	5 (10%)

*Multiple categories possible for each article, thus may not total 100%.

The bolded numbers indicate the number of studies that relate to that particular focus/subheading. So, the subvariables under each subheading are a proportion of the total bolded number for each subheading.

Evidence-based practices and recommendations for developing competency frameworks and for building competencies within public health students and practitioners are identified (Table 4). Of the studies that identified evidence-based practices and approaches, the need for governance and resources to oversee competency framework development, refreshing, implementation across the workforce, and evaluation are included (18%). Multi-step and consensus-building approaches (14%) are recommended by studies that focused on building competency statements and frameworks, followed by a call for additional approaches to address complex public health issues (e.g., health inequities, Indigenous health). Experiential learning (26%), including practicum and community-based learning, was found to be best for developing competencies in public health students and practitioners. Matching training curricula to competencies (23%) was also found to be an effective practice for developing competencies in public health students and practitioners.

**Table 4:** Evidence-based practices and recommendations for developing competencies in public health.

Evidence-based practices and approaches (*n* = 96/120, 80%)	*n* (%) *n*/96 (100%)
Resources and governance needed	17 (18%)
Developing competency statements and frameworks*	
Multi-step and consensus-building approaches best for developing competency statements	13 (14%)
Additional approaches to look at competencies to address complexity needed	9 (9%)
Validation instruments to measure competency development as a result of training needed	5 (5%)
Tension between the need for foundational competency statements and discipline- and/or expertise-specific statements	5 (5%)
Evaluation of the utility and impact of competency frameworks needed	4 (4%)
Refreshing competency frameworks required on a regular schedule	2 (2%)
Increased transparency in methods and reporting within competency development research needed	2 (2%)
Developing competencies within students and practitioners*	
Experiential learning is useful for developing competencies	25 (26%)
Matching curriculum to competencies necessary	22 (23%)
Regular tiered (e.g., basic to advanced) training responsive to current public health needs are required to build competencies	11 (11%)
Mentorship between experienced and new public health practitioners or students reduces barriers, creates a professional network, and provides feedback	8 (8%)

*Multiple categories possible for each article, thus may not total 100%.

## 4 Discussion

Effective public health practice requires a skilled interdisciplinary public health workforce. Competencies describe the integrated knowledge, values, skills, and behaviors required for practitioners and organizations across the practice of public health (1, 8, 10, 16, 32). Competency frameworks allow for workforce planning and development, including identifying gaps in training, evaluating performance, and developing professional development opportunities (32). Key components in developing the public health workforce include developing competency-based curricula and a comprehensive and coordinated lifelong learning system (33). Understanding successes, shortfalls, and lessons learned from previous literature on competency development is important for developing a strong public health workforce going forward. However, the lack of consistency in methods across the body of literature on this topic, the focus on foundational competencies and general practitioners, along with limited reporting on best practices, may make it challenging for public health researchers and organizations to develop competencies effectively and efficiently within the workforce.

The included literature was divided into literature that describes how competency statements and frameworks have been developed and refreshed (*n* = 13, 11%) and literature describing how to develop competencies within public health students and practitioners (*n* = 107, 89%). Studies were most often mixed methods research conducted in the United States by academia and spanned various target populations within public health students and professionals. Most of the included literature focused on building competencies through student and professional development, with the former most often offered through academic courses and the latter most often offered online. Surveys, including pre- and post-surveys, are used for self-assessment of competency development among students and practitioners. The included literature had a limited focus on approaches and best practices for developing competency statements and frameworks. Within the relevant literature, experiential and adult learning were the most applied pedagogical approaches.

### 4.1 Competency literature focused on a general public health audience and foundational competencies

The focus on foundational and core competencies is necessary to provide the cross-cutting knowledge, values, and skills integrated by a practitioner to perform broad functions across public health (34). These studies provide the basis by which practitioners and students can engage in critical thinking and reflection and allow for the development of more advanced and discipline-specific knowledge, values, skills, and behaviors (35). Thus, as more is understood about developing core competency frameworks and foundational competencies across the workforce, discipline-specific competency frameworks and training programs that support depth in knowledge, skills, and behaviors can be built more efficiently.

Specific public health audiences varied across the included literature, with the majority focusing on general public health practitioners. The public health workforce is comprised of practitioners who engage in public health work as the primary part of their role (33). Within the public health workforce, there are diverse disciplines and professional backgrounds that require unifying competencies and professional development to ensure optimal individual and organizational performance (14). A focus on general public health practitioners provides the evidence-base required for understanding and applying best practices for workforce development across the public health system.

Similarly, most of the literature focused on approaches for developing foundational or core competency frameworks, and professional development across foundational competencies. There was an overall lesser focus on professional development on the topics of cultural competency, evidence-informed decision-making, crisis and risk communication, epidemiology, and public health leadership. However, discipline-specific competencies, coupled with foundational competencies are necessary to address complex and multifaceted public health issues including social determinants of health, health equity, and climate change (36, 37).

### 4.2 Gaps exist in competency research including a limited focus on discipline-specific competency research

The modest amount of discipline-specific competency research uncovered in the current review was mainly in the realm of professional development, including a focus on health equity, health literacy, epidemiology, evidence-informed decision-making, crisis and risk communication, and cultural competency. System-level improvement requires developing and maintaining a trained public health workforce. Key aspects of providing effective professional development include mapping competencies to curricula and understanding training gaps in the workforce (38). A recent study identified communication, change management, budgeting, and reporting as gaps in the U.S. public health workforce (39). Similarly, communication was identified as a gap among Israeli public health practitioners, along with responding to current and emerging public health issues (38). However, studies examining competency gaps in public health practitioners at the country level are limited. An understanding of the strengths and gaps in public health competencies both today and in the future is required to be able to map professional development opportunities, such as in public health communication, to strengthen the workforce.

Further, few included studies focused on discipline-specific competency statements and framework development. Both foundational and discipline-specific competencies are necessary to provide the cross-cutting knowledge, values, skills, and behaviors necessary for core public health functions. Public health encompasses a range of disciplines and backgrounds and requires not only a grounding foundational set of competencies, but also discipline-specific competencies used by public health specialists (12). Key public health disciplines requiring a specialized competency framework through which the foundational competencies are integrated include epidemiology, infectious disease, global health, nursing, communication, and others (40). Additional literature on developing discipline-specific competency frameworks, including health communication competencies, and competency-based training would add to our understanding of additional professional development and workforce planning requirements across the public health workforce.

Within the literature on developing competency statements and frameworks, gaps were also identified related to the trustworthiness of the research. More than half the included literature emphasized the need for validated instruments to measure change in competencies as a result of education or professional development to increase the trustworthiness of research. Further, studies reporting on competency statements and framework development should aim to increase their transparency to allow for replication and the ability to better contextualize and understand the results. Recent recommendations for reporting competency framework development in health professions (CONFRED-HP) echo this as demonstrated by the inclusion of a number of key items related to transparency in their checklist (41). Finally, evaluation of the utility and impact of competency frameworks is lacking and should be undertaken to better understand how to develop frameworks that meet the needs of public health organizations. Very few evaluations of public health competency frameworks exist, but those that do provide an important contribution to related literature and workforce development (42). Evaluation and implementation are also areas of focus within the CONFRED-HP checklist to support this key recommendation (41).

### 4.3 Evidence-based practices and approaches for competency development point toward a need for resources, governance, and real-world application

Several best practices and approaches for public health competency development were found in the included literature. The need for resources and governance to oversee competency framework development, revitalization, and workforce planning was commonly identified. Governance of competency frameworks by federal governments is needed to provide oversight and resources for public health workforce planning and development and to ensure equal access to an effective workforce for all communities. In Canada, the Canadian Public Health Association and others have called on the federal government to create a new Public Health Act that supports public health, oversees competency framework development and implementation, and provides resources to the system (14, 43). In the United States, the Council on Linkages between Academia and Public Health Practice is comprised of 24 national organizations that collaborate to support public health competence development in the workforce (44). Governance ensures competency frameworks stay up to date, are incorporated into practice, are adequately financed, and are mapped to professional development opportunities. Ensuring the public health workforce has modern competencies to fulfill its functions requires dedicated resources and an interdisciplinary and collaborative governance structure to oversee the renewal, implementation, and evaluation of competency frameworks.

Multi-step and consensus-building approaches for developing competency statements and frameworks in public health are identified as best practices by all the related included literature. The Delphi technique is a method often used for competency framework development to gain consensus from a group of experts (45). Delphi techniques contain a number of rounds that may include expert consultation, surveys, interviews, and focus groups, depending on the literature in the area and the number of rounds required for consensus or agreement. There are no specific recommendations on the number of rounds or the order of methods included in Delphi technique; however, it is clear that a multi-step process that builds consensus is key to ensure rigor (45, 46).

Within competency development for public health students and practitioners, experiential learning and curriculum-based training were commonly identified as evidence-based practices. Experiential learning provides real-world public health experiences as a means of developing competencies beyond the classroom and is often incorporated in practicums, capstone courses, and sometimes within individual courses and assessments (47, 48). Experiential learning centers practical experiences within the education, allowing for the integration of knowledge, values, skills, and behaviors (47). Further, competency-based training embeds competencies within the curricula, assessments, and structure of the training opportunity. Competency-based education and training complements experiential learning through orienting the application of knowledge, values, skills, and behaviors in the real world (49). Importantly, it also accelerates graduate’s to job readiness, thereby enhancing public health capacity. The focus on the real-world application of competency-based education and training and experiential learning bridges the gap between the public health workplace, evidence, and the education or training setting (49).

### 4.4 Limitations and future research

With regards to the included literature, selection bias and generalizability of results were identified within the included literature as the most common biases. These biases can threaten the validity of the research and impact the generalizability of findings.

Within the methods used to conduct the scoping review, limitations also exist including the selection of databases. Language bias is also present as included articles needed to be in English.

Future research using robust mixed methods to assess how competencies are developed and the best instrument to measure changes in competencies reliably and efficiently is needed. Additional research on developing, implementing, and evaluating discipline-specific competency frameworks is also needed. With regards to experiential learning, additional evaluation of various opportunities in public health should be conducted to increase the validity of findings and contribute to an increased understanding of best practices.

## 5 Conclusion

This scoping review highlights the importance of competency frameworks and professional development in improving public health practice and developing a skilled, interdisciplinary public health workforce. Competency frameworks are essential tools for workforce planning and development, including identifying training gaps, evaluating performance, and creating professional development opportunities. Much of the included literature focused on foundational competency framework development and professional development for general public health practitioners. While this provides an important broad understanding of the essential competencies needed, there is a gap in discipline-specific research. Public health is a diverse field comprising a range of disciplines that require competency development and frameworks for specialized areas of practice. This review also highlights several best practices for competency development, including the importance of governance and resources to oversee competency framework development and workforce planning, as well as experiential learning and competency-based training for students and practitioners.

## Author contributions

MM: Conceptualization, Data curation, Formal analysis, Funding acquisition, Investigation, Methodology, Software, Supervision, Validation, Visualization, Writing – original draft, Writing – review & editing. CF: Writing – review & editing, Formal analysis, Investigation, Validation. LG: Funding acquisition, Validation, Writing – review & editing. AP: Conceptualization, Funding acquisition, Validation, Writing – review & editing. JM: Writing – review & editing, Conceptualization, Funding acquisition, Methodology, Project administration, Resources, Supervision.
