# Supplementary material for: Developing competencies in public health: a scoping review of the literature on developing competency frameworks and student and workforce development
Source: Front Public Health. 2024 Mar 4;12:1332412. doi: 10.3389/fpubh.2024.1332412 (PMC10944919; doi:10.3389/fpubh.2024.1332412)
Supplement: Supplementary file 1 [file Data_Sheet_1.PDF]

Supplementary Table 1: Full Listing of All Included Literature in Scoping Review (n=120)

Author(s)	Year published	Article title	Study aim/purpose	Methods type	Document type -> Journal article	Document type -> Grey Literature	Brief Methodology
Emma Apatu, Deborah M. Owen, Sericea Stallings-Smith, Aaron Spaulding, Hanadi Hamadi	2020	Visit to the World Health Organization: student perceptions of interprofessional learning after a short-term public health study abroad course in Switzerland.	Describing interprofessional learning from a short-term study abroad PH course	Mixed methods	Journal article		Mixed methods, pre- post- assessment
O'Neall, M A, Brownson, R C	2005	Teaching evidence-based public health to public health practitioners.	Describing and evaluating evidence-based public health course	Qualitative	Journal article		Descriptive, workshop developed, delivered by many organizations and evaluated
Porterfield, D S, Marcial, L H, Brown, S, Throop, C, Pina, J	2017	Evaluation of a quality improvement resource for public health practitioners: the Public Health Quality Improvement Exchange.	Evaluate the Public Health Quality Improvement Exchange	Mixed methods	Journal article		Survey of PHQIX users
Mabhala, M A	2013	Health inequalities as a foundation for embodying knowledge within public health teaching: a qualitative study.	to improve the training of PHNEs by identifying gaps in PHNE's knowledge and experience that need to be filled	Qualitative	Journal article		Qualitative in-depth semi-structured interviews
Hawley, S R, St. Romain, T, Rempel, S L, Orr, S A, Molgaard, C A	2012	Generating social capital through public health leadership training: a six-year assessment.	determining the program's quantitative influence on generating social capital, as well as describing the qualitative benefits of this increased social capital in participants' professional environment	Quantitative	Journal article		pre- post- assessment of training
Mazzucca, S, Jacob, R R, Valko, C A, Macchi, M, Brownson, R C	2022	The relationships between state health department practitioners' perceptions of organizational supports and evidence-based decision-making skills.	To quantify perceptions of individual EBDM skills and Administrative-EBPs, as well as the longitudinal associations between the 2	Quantitative	Journal article		Repeated survey at 2 points in time
Hughes, R	2003	Competency development in public health nutrition: reflections of advanced level practitioners in Australia	Investigate attitudes, experiences, beliefs of advanced level PH nutritionists in relation to PH nutrition competency development.	Qualitative	Journal article		Qualitative using semi-structured interviews
Baukus, A J	2019	Developing a Community-Based Research Project Proposal to Build Public Health Educator Capacity: A Graduate Student Perspective	Author's first-person perspective of completing an Applied Practice Experience (APE) at an accredited public health program	Qualitative	Journal article		Personal reflection

Schnall, A, Nakata, N, Talbert, T, Bayleyegn, T, Martinez, D, Wolkin, A	2017	Community Assessment for Public Health Emergency Response (CASPER): an innovative emergency management tool in the United States.	demonstrate how inclusion of the Centers for Disease Control and Prevention’s Community Assessment for Public Health Emergency Response (CASPER) as a tool in Public Health Preparedness Capabilities: National Standards for State and Local Planning can increase public health capacity for emergency response	Quantitative	Journal article	Reviewed and compared CASPER activities incl. efficacy and usefulness
Wilson, K, Juya, A, Abade, A, Sembuche, S, Leonard, D, Harris, J, Perkins, A, Chale, S, Bakari, M, Mghamba, J, Kohler, M	2021	Evaluation of a new field epidemiology training program intermediate course to strengthen public health workforce capacity in Tanzania	Field epidemiology training program course aims to strengthen health workforce capacity in surveillance system assessment, outbreak investigation, and evaluation	Mixed methods	Journal article	pre/post evaluation design using data from 4 cohorts of trainees who took the course
Olson, D, Hoeppner, M, Larson, S, Ehrenberg, A, Leitheiser, A T	2008	Lifelong learning for public health practice education: a model curriculum for bioterrorism and emergency readiness.	Describing how the University of Minnesota School of Public Health developed and implemented a tool for lifelong learning model for bioterrorism and emergency readiness	Qualitative	Journal article	Modified delphi needs assessment, implementation, evaluation
O'Connell, E, Stoneham, M, Saunders, J	2015	Planning for the next generation of public health advocates: evaluation of an online advocacy mentoring program.	Evaluating an online e-mentoring program for public health professionals to gain knowledge through skill-based activities and engaging in a mentoring relationship with an experienced public health advocate	Qualitative	Journal article	semi-structured interview qualitative evaluation of the online e-mentoring program.
Bondy, S J, Johnson, I, Cole, D C, Bercovitz, K	2008	Identifying core competencies for public health epidemiologists.	Seeking perspectives on important competencies among epidemiologists familiar with or practicing in public health settings (local to national).	Mixed methods	Journal article	Interviews, consensus building workshop,
Mainor, A G, Decosimo, K, Escoffrey, C, Farris, P, Shannon, J, Winters-Stone, K, Williams, B, Leeman, J	2018	Scaling up and tailoring the "Putting Public Health in Action" training curriculum.	describe the training curriculum and findings from the Network’s evaluation of approaches used to scale up delivery of the “Putting Public Health Evidence in Action” curriculum and tailor content for specific evidence-based interventions	Mixed methods	Journal article	cross-sectional survey design with participants following training
Hearne, S A	2008	Practice-based teaching for health policy action and advocacy.	describes a potential model curriculum for introductory health-advocacy theory and skills based on the course	Qualitative	Journal article	Describing a program
Lenthall, S, Wakerman, J, Knight, S	2009	The frontline and the ivory tower: a case study of service and professional-driven curriculum.	describe the development of a postgraduate, multidisciplinary program designed to meet the needs of remote health professionals, present formative evaluation findings and to offer an analysis of the difficulties and lessons learnt.	Qualitative	Journal article	case-study format, describing the program's development
Wright, J, Mala, Rao, Walker, K	2008	The UK Public Health Skills and Career Framework - could it help to make public health the business of every workforce?	to describe how the UK Public Health Skills and Career Framework was developed, and to invite discussion on its potential usefulness as a tool for facilitating a shared approach to strengthening public health competence within and across countries.	Qualitative	Journal article	Qualitative, consult colleagues and agree with bottom-up approach

Dilley, J A, Reuer, J R, Colman, V, Norman, R K	2009	Steps to a healthier Washington: from making pamphlets to making policies: results from a collaborative training to increase knowledge, motivation, and self-efficacy for achieving public health policy and systems change.	Describing and evaluating a to training to improve public health practice and create greater organizational and staff capacity for promoting effective policy and systems changes	Mixed methods	Journal article	program description and evaluation using pre- post exams and satisfaction surveys
Woodard, LeChauncy, Liaw, Winston, Adepoju, Omolola E, Prabhu, Sahana, Chae, Minji, Matuk-Villazon, Omar, Beech, Bettina M	2022	Evaluating a contact tracing course: How universities can develop the public health workforce.	describe the participants of a university-based COVID-19 contact tracing course and determine whether the course changed knowledge, attitudes, and intention to participate in contact tracing.	Quantitative	Journal article	Surveys to evaluate course impact and subsequent analysis
Chan, Linda, Mackintosh, Jeannie, Dobbins, Maureen	2017	How the “Understanding Research Evidence” Web-Based Video Series From the National Collaborating Centre for Methods and Tools Contributes to Public Health Capacity to Practice Evidence-Informed Decision Making: Mixed-Methods Evaluation	The first four videos in the URE web-based video series, which explained odds ratios (ORs), confidence intervals (CIs), clinical significance, and forest plots, were evaluated. The evaluation examined how the videos affected public health professionals’ practice.	Mixed methods	Journal article	A three-part evaluation was conducted to determine the effectiveness of the first four URE videos. The evaluation included a Web-based survey, telephone interviews, and pretest and posttests,
Meagher-Stewart, Donna, Solberg, Shirley M, Warner, Grace, MacDonald, Jo-Ann, McPherson, Charmaine, Seaman, Patricia	2012	Understanding the role of communities of practice in evidence-informed decision making in public health.	consensus-building workshops were to explore first, how public health practitioners understood, defined, and used EIDM and CoP, and second, how CoP facilitated EIDM to enhance their practice.	Qualitative	Journal article	Consensus building workshops to gain insight into PH practitioners' shared understanding and use of communities of practice and EIDM
Dickson, Michelle, Manalo, Giselle	2014	The Beyond Borders Initiative: Aboriginal, Torres Strait Islander and international public health students: Engaging partners in cross-cultural learning.	To increase student knowledge about PH issues and current health promotion practices in Australian Aboriginal and Torres Strait Islander communities	Mixed methods	Journal article	implementation of a community-oriented curriculum
Zahner, Susan J, Henriques, Jeffrey B	2014	Public health practice competency improvement among nurses.	measure nurses' competency for public health practice at baseline, identify factors associated with higher competency, document change in competency over 4 years, and assess differential effects associated with project participation.	Quantitative	Journal article	ompetency was assessed using a validated instrument administered through online surveys at baseline (2007) and 4-year follow-up (2011)
Wood, Annette	2015	Learning, assessment and professional identity development in public health training.	To assess the integration of knowledge and assessment in PH training programs	Qualitative	Journal article	Semi structured interviews, questionnaires and thematic analysis
Beaton, Randal D, Johnson, L Clark, Maida, Carl A, Houston, J Brian, Pfefferbaum, Betty	2012	Disaster Research Team Building: A Case Study of a Web-based Disaster Research Training Program.	describes the process and outcomes of the Northwest Center for Public Health Practice Child and Family Disaster Research Training (UWDRT) web-based Program	Qualitative	Journal article	Case study describing course development, curriculum refinement evaluation and outcomes

Morshed, A B, Ballew, P, Elliott, M B, Haire-Joshu, D, Kreuter, M W, Brownson, R C	2017	Evaluation of an online training for improving self-reported evidence-based decision-making skills in cancer control among public health professionals.	assess the effect of the online evidencebased cancer control (EBCC) training on improving the self-reported evidence-based decision-making (EBDM) skills in cancer control among Nebraska public health professionals.	Quantitative	Journal article	Cross-sectional group comparison, surveys to compare skill difference with those completed training/baseline
Fifolt, Matthew, McCormick, Lisa C, Carvalho, Michelle, Lloyd, Laura, Alperin, Melissa	2020	Connecting public health students to rural and underserved areas: Promoting health equity through field placement experiences.	To describe PH student experiences in the PH Training Centers Pathways to Practice ScholarsProgram	Mixed methods	Journal article	Sequential, exploratory mixed methods design, pre and post confidence scores and surveys, thematic analysis
Hawley, Suzanne R, Crimmings, Karen P, Rivera-Newberry, Ivonne, Orr, Shirley A, Walkner, Laurie M	2020	Capacity building for public health: Participant-guided training.	To describe development of a diabetes intervention training summit and its evaluation.	Mixed methods	Journal article	Gap analysis, program planning, implementation, evaluation
Shi, Lei, Fan, Lihua, Xiao, Hai, Chen, Zhenkang, Tong, Xinfa, Liu, Ming, Cao, Depin, Lei, Shi, LiHua, Fan, Hai, Xiao, ZhenKang, Chen, XinFa, Tong, Ming, Liu, DePin, Cao	2019	Constructing a general competency model for Chinese public health physicians: a qualitative and quantitative study.	to construct a general model of the competencies required by Chinese public health physicians	Mixed methods	Journal article	Mixed methods, interviews and expert consultations
Bell, Marnie, MacDougall, Karen	2013	Adapting online learning for Canada's Northern public health workforce.	To assess the appropriateness of the PHAC Skills Online program for Northern/Aboriginal public health workers	Mixed methods	Journal article	measures to assess relevancy, effectiveness and design and delivery of the pilots. Data sources included focus groups, online module feedback surveys, facilitators' journal log and notes, Pilot Planning Group feedback and one-on one telephone
Poulton, Brenda, Lyons, Agatha, O'Callaghan, Anne	2008	A comparative study of self-perceived public health competencies: practice teachers and qualifying SCPHNs.	To compare nursing practice teachers self-assessed knowledge and skills in 10 areas to those of student PH nurses	Quantitative	Journal article	Quantitative questionnaire
Baseman, Janet G, Marsden-Haug, Nicola, Holt, Victoria L, Stergachis, Andy, Goldoft, Marcia, Gale, James L	2008	Epidemiology competency development and application to training for local and regional public health practitioners.	developing competency-based epidemiology training for non-epidemiologist public health practitioners in the northwestern United States.	Quantitative	Journal article	Qualitative, Development of competencies, implementation in curriculum, evaluation of the programs
Lederer, Alyssa M, Barrett, Karla Todd, Shorter, Charles, Kenefick, Hope W, Kulik, Phoebe K G, Morales, Marcia, Reinschmidt, Kerstin M, Shrestha, Sweta	2022	Public Health Training Centers' Support for Community Health Workers: Case Studies of Needs Assessment, Training, and Student Field Placement Initiatives.	To describe and evaluate the role of Public Health Training Centres (PHTCs)	Quantitative	Journal article	Case studies from 3 PHTCs are provided to exemplify how PHTCs are well positioned to support the critical CHW workforce via assessment, training, and student field placements.
Ablah, Elizabeth, Nickels, Debbie, Hodle, Amanda, Wolfe, Deborah J, Orr, Shirley, Tenbrink, Jerry, Ploger-McCool, Terri, Snethen, Edie, Molgaard, Craig A	2007	"Public health investigation": a pilot, multi-county, electronic infectious disease exercise.	Describes the quantitative evaluation of a month-long, multi-county, real-time infectious disease pilot exercise that was conducted using electronic media, which allowed participants to work from their health department offices as they would in a real incident	Quantitative	Journal article	Quantitative evaluation of program using pre- and postsureys

Zwanikken, Prisca A C, Alexander, Lucy, Scherpbier, Albert	2016	Impact of MPH programs: contributing to health system strengthening in low- and middle-income countries?.	Describing qualitative findings of two English language programs, one a distance MPH program offered from South Africa, the other a residential program in the Netherlands. Both offer MPH training to students from a diversity of countries. In-depth interviews were conducted with 10 graduates (per program), working in low- and middle-income health systems, their peers, and their supervisors	Qualitative	Journal article		Interviews with PH students/graduates
Dos Santos, L M	2019	Rural Public Health Workforce Training and Development: The Performance of an Undergraduate Internship Programme in a Rural Hospital and Healthcare Centre	The research examined the performance, feedback, and opinions of a university-based one-year-long on-site internship training programme between a university public health and healthcare undergraduate department and a regional hospital and healthcare centre in a rural region in the United States.	Qualitative	Journal article		Individual interview data were collected from management trainees and focus group activities data were collected from hospital departmental supervisors who have completed this one-year-long on-site internship training programme
Hawley, S R	2020	Using adaptive leadership principles to support Public Health 3.0 in multidisciplinary undergraduate education	This paper aims to provide an educational framework for implementing adaptive leadership instruction for undergraduate students.	Quantitative	Journal article		Describe and evaluate, pre and post surveys
Biddinger, P D, Savoia, E, Massin-Short, S B, Preston, J, Stoto, M A	2010	Public Health Emergency Preparedness Exercises: Lessons Learned	Harvard School of Public Health Center for Public Health Preparedness exercise program has two aims: (1) educating the public health workforce on key public health system emergency preparedness issues, and (2) identifying specific systems-level challenges in the public health response to large-scale events	Quantitative	Journal article		Evaluation of exercise program using surveys, post-exercise evaluation,
Plugge, E, Cole, D	2011	Oxford graduates' perceptions of a global health master's degree: a case study	To describe and evaluate the global health master's degree	Qualitative	Journal article		Case study, interviews conducted with program graduates
Allegrante, J P, Barry, M M, Airhihenbuwa, C O, Auld, M E, Collins, J L, Lamarre, M C, Magnusson, G, McQueen, D V, Mittelmark, M B, Conference, Galway Consensus	2009	Domains of Core Competency, Standards, and Quality Assurance for Building Global Capacity in Health Promotion: The Galway Consensus Conference Statement	reports the outcome of the Galway Consensus Conference, an effort undertaken as a first step toward international collaboration on credentialing in health promotion and health education	Qualitative	Journal article		Qualitative, conference proceedings to reach agreement
Yost, J, Mackintosh, J, Read, K, Dobbins, M	2016	Promoting Awareness of Key Resources for Evidence-Informed Decision-making in Public Health: An Evaluation of a Webinar Series about Knowledge Translation Methods and Tools	To inform continued implementation of NCCMT Spotlight on KT Methods and Tools webinar series, NCCMT conducted an evaluation of the series' potential to improve awareness and use of the methods/tools within the Registry, as well as identify areas for improvement and "what worked."	Mixed methods	Journal article		electronic follow-up surveys administered immediately following each webinar; an additional electronic survey administered 6 months after two webinars; and Google Analytics for each webinar.

Perez, J, Leonard, W R, Bishop, V, Neubauer, L C	2021	Developing Equity-Focused Education in Academic Public Health: A Multiple-Step Model	A four-step Curricular Responsive Review Model (CRRM) generated by educators in a Council on Education for Public Health–accredited public health program to systematically understand and gather feedback on how to develop a responsive curriculum for their students.	Mixed methods	Journal article		Review course syllabi, engage with community, incorporate findings into curricula
Welter, C R, Jarpe-Ratner, E, Xu, D N, Fouche, S, Naji, S, Bisesi, M	2020	Increasing Environmental Public Health Practitioner Capacity to Address Population Health Challenges: Evaluation Results From a Workforce Development Project	An evaluation of an environmental PH practitioner workforce development initiative	Mixed methods	Journal article		A multicomponent evaluation, incorporating post-session satisfaction surveys, action learning discussion transcripts, post-session refl ections, and follow-up interviews at 6 months were completed.
Rusnak, L, Peek, J T, Orriola, D, Makut, M B	2019	Integrating Diverse Study Abroad Opportunities Into Public Health Curricula: Three Distinct Strategies to Address Common Barriers	This manuscript provides strategies to integrate diverse study abroad programming into public health curriculum and mitigate common barriers for students and faculty	Mixed methods	Journal article		used three strategies to improve access to global learning: 1. Adding public health courses to established study abroad programs 2. Utilizing academic travel companies 3. Leveraging existing international and university partnerships.
Risisky, D, Goldson, T, DeMezzo, R	2020	Post-Graduation Impact of a Program Planning Service-Learning Project	evaluation of an undergraduate public health course sought to understand the impact of service-learning on material retention, internships, and post-graduation public health careers.	Mixed methods	Journal article		Qualitative, open ended questionnaire
Riley-Jacome, M, Parker, B A G, Waltz, E C	2014	Weaving Latino Cultural Concepts Into Preparedness Core Competency Training	This article describes initiatives undertaken by the NY•NJ PERLC to improve the capacity of the public health workforce to respond competently to the needs of Latino populations.	Mixed methods	Journal article		Description of the programs implemented and an evaluation via participant surveys
Dewa, Carolyn S, Che, Zoe, Guggenbickler, Andrea M, Phan, Rebecca, Pollock, Bradley	2022	Building a public health workforce for a university campus during a pandemic using a practicum framework: Design and outcomes.	This paper describes the use and outcomes of a practicum framework to quickly create a university-based public health workforce. It addresses two questions: (1) Using a practicum framework, what are important considerations in designing and building a public health workforce for a university campus? and (2) What are the benefits to the workforce in terms of public health education and professional growth?	Mixed methods	Journal article		Program administrative data were used to describe the workforce and their learning outcome
Tshitangano, Takalani G	2016	An exploratory study of the need for curriculum review of Master of Public Health Degree at a Rural-based University in South Africa.	To determine whether the MPH programme at the selected rural-based university in South Africa enabled students to achieve the MPH core competencies relevant for Lower Middle Income Countries.	Quantitative	Journal article		A quantitative cross-sectional descriptive research design with questionnaires
Kahn, K, Tollman, S M	1992	Planning professional education at schools of public health.	Describing and review of Harvard School of PH self evaluation and development of practice-oriented program	Quantitative	Journal article		Self administered questionnaires and discussion with students/faculty

Yarber, Laura, Brownson, Carol A, Jacob, Rebekah R, Baker, Elizabeth A, Jones, Ellen, Baumann, Carsten, Deshpande, Anjali D, Gillespie, Kathleen N, Scharff, Darcell P, Brownson, Ross C	2015	Evaluating a train-the-trainer approach for improving capacity for evidence-based decision making in public health.	This study examines the outcomes achieved among participants of courses led by trained state-level faculty	Quantitative	Journal article	Surveys administered to participants
Norton, Wynne E	2014	dvancing the science and practice of dissemination and implementation in health: a novel course for public health students and academic researchers.	This article describes the D&I course in terms of content, format, participants, and the collaborative learning project; presents findings from the online course evaluation; and suggests viable strategies for adapting and implementing this course at other academic institutions.	Mixed methods	Journal article	Description of program and brief evaluation
Armstrong-Mensah, Elizabeth, Ramsey-White, Kim, Alema-Mensah, Ernest	2019	Integrative Learning in US Undergraduate Public Health Education: A Review of Student Perceptions of Effective High-Impact Educational Practices at Georgia State University.	This review seeks to examine student perceptions of integrative practices utilized by Georgia State University faculty in the BSPH program and to investigate the extent to which student perceive these integrative educational practices as preparing them to use insights gained in the classroom and from the field, to question, modify, connect, and integrate material learned in the academic setting, to real-life public health challenges. It also seeks to identify which of the integrative educational practices have the highest impact of helping students integrate the knowledge and skills gained to public health issues.	Quantitative	Journal article	Cross sectional, surveys sent to current students
Bourgeois, I, Simmons, L, Buetti, D	2018	Building evaluation capacity in Ontario's public health units: promising practices and strategies.	This article presents the findings of a project focusing on building evaluation capacity in 10 Ontario public health units. The study sought to identify effective strategies that lead to increased evaluation capacity in the participating organizations.	Mixed methods	Journal article	This study used a qualitative, multiple case research design.
Heading, Gaynor S, Fuller, Jeffrey D, Lyle, David M, Madden, D Lynne	2007	Using problem-based learning in public health service based training.	This study explored the suitability and benefits of problem-based learning (PBL) in competency-based postgraduate public health training. The PBL was delivered within a rural retreat and included site visits.	Qualitative	Journal article	Qualitative semistructured interviews with trainee public health officers and key informants
Parker, Shan, Johnson-Lawrence, Vicki	2022	Addressing Trauma-Informed Principles in Public Health through Training and Practice.	This study examined trauma-informed principles and related terminology for use in public health coursework in the context of a community-wide water contamination public health crisis in Flint, Michigan, USA.	Qualitative	Journal article	Description of the development of the trauma-informed PH course (no evaluation)

D'Ambrosio, Luann, Huang, Claire E, Sheng Kwan-Gett, Tao	2014	Evidence-based communications strategies: NWPERLC response to training on effectively reaching limited English-speaking (LEP) populations in emergencies.	This report describes how a Preparedness and Emergency Response Learning Centre (PERLC) and a Preparedness and Response Research Centre (PERRC) colocated at the Northwest Center for Public Health Practice responded to Los Angeles County Department of Public Health's (DPH's) request to improve emergency communications with limited English-proficient (LEP) populations	Mixed methods	Journal article	Activities included an assessment of training needs of the DPH preparedness workforce, a training series on social media and community engagement, and a toolkit of evidence-based findings to improve LEP populations' emergency communications and community resilience. R
Montgomery, JoLynn P, Durbeck, Heidi, Thomas, Dana, Beck, Angela J, Sarigiannis, Amy N, Boulton, Matthew L	2010	Mapping student response team activities to public health competencies: are we adequately preparing the next generation of public health practitioners?.	This article compares activities of the University of Michigan School of Public Health Public Health Action Support Team (PHAST) to the Centers for Disease Control and Prevention/Council of State and Territorial Epidemiologists Applied Epidemiology Competencies (AECs) to determine the utility of using the competencies to assess extracurricular student training.	Mixed methods	Journal article	mapped the activities from eight PHAST trips occurring to the 34 AECs by examining project activities to determine how closely they aligned with the AECs
Belliard, Juan Carlos, Dyjack, David T	2004	Putting the Public into the public health curriculum: a case study.	This case study focuses on the educational outcomes of a pilot academic service-learning course that is taught in the Global Health Department of Loma Linda University's School of Public Health.	Qualitative	Journal article	Description of course structure and reflection from students
Palermo, Claire, Hughes, Roger, McCall, Louise	2010	A qualitative evaluation of an Australian public health nutrition workforce development intervention involving mentoring circles.	To evaluate a mentoring circle workforce development intervention among a group of public health nutrition novices.	Qualitative	Journal article	mentoring circle intervention, retrospective post-intervention qualitative semi-structured interview
McIntosh, Scott, Perez-Ramos, Jose G, David, Tamala, Demment, Margaret M, Avendano, Esteban, Ossip, Deborah J, De Ver Dye, Timothy	2017	A globally networked hybrid approach to public health capacity training for maternal health professionals in low and middle income countries.	To report on the first year results of the MundoComm project -- an NIH funded project for sustainable public health capacity building in community engagement and technological advances aimed at improving maternal health issues.	Mixed methods	Journal article	quantitative and qualitative feedback (using online data capturing forms) assess baseline and posttraining knowledge and skills in public health project strategies.
Matthews & Jackson	2021	Application of a return of investment analysis for public health training by case study	We utilized the use case of HAI outbreaks associated with the Legionella pathogen to provide metrics for our proforma model to make the business case that investing in training specifically the CIC was worthwhile for cost reduction, improved KSA's and improved retention	Qualitative	Journal article	In this 2018 case study analysis, we coupled the Phillip's Return on Investment model with a standard financial proforma model to make a business case that investing in training, specifically the Certification in Infection Control (CIC), was worthwhile for cost reduction, improved knowledge, skills and abilities (KSA's) and improved employee retention.
Salinas-Miranda, Nash, Salemi, Mbah, & Salihu	2013	Cutting-edge technology for public health workforce training in comparative effectiveness research	The purpose of this case study is to describe the development and formative evaluation of a technologyenhanced comparative effectiveness research learning curriculum and to assess its potential utility to improve core comparative effectiveness research competencies among the public health workforce.	Qualitative	Journal article	We used a single-case study design11 to illustrate the operational framework of a blended learning CER training program, namely, e-CER (pilot intervention), and a modified Delphi evaluation (formative evaluation) that integrated a variety of informatics and communication tools.

MacVarish, Kenefick, Fidler, Cohen, Orellana & Todd	2018	Building Professionalism Through Management Training: New England Public Health Training Center's Low-Cost, High-Impact Model	Evolving practices, accreditation, and priorities established in Public Health 3.0 are adding to the long-identified need for management training among public health practitioners.	Mixed methods	Journal article	addressing this need with a flexible, open-source, 16-topic training program. The program is designed to build competencies for current and future managers, preparing them for their day-to-day tasks and for the kinds of adaptation suggested by Public Health 3.0
Caira, Lachenmayr, Sheinfeld, Goodhart, Cancialosi, Lewis	2003	The Health Educator's Role in Advocacy and Policy: Principles, Processes, Programs, and Partnerships	This article provides a "how-to" guide for health education practitioners by showcasing five initiatives undertaken by health educators in New Jersey to promote the use of advocacy.	Qualitative	Journal article	Recognizing this as a challenge and opportunity, NJ SOPHE and NJPHA collaborated in 1998 to develop what has become a series of advocacy training workshops for public health professionals.
Wright, Hann, McLeroy, Steckler, Matulionis, Auld, Lancaster, & Weber	2003	Health Education Leadership Development: A Conceptual Model and Competency Framework	Conceptual model and competency framework for PH education leadership institute.	Qualitative	Journal article	Conceptual framework and model developed through needs assessment with senior-level health educators to establish program needs.
Qureshi et al.	2004	Effectiveness of an Emergency Preparedness Training Program for Public Health Nurses in New York City	To report on the impact of a training program designed to prepare public health nurses to respond appropriately to emergencies.	Quantitative	Journal article	To develop a training program, pilot test the program with 50 public health nurses, conduct two sessions of the program, and evaluate.
Farel, Umble & Pohamus	2001	Impact of an Online Analytic Skills Course	COURSE This article describes the effect of an online analytic skills training course on professional development and practice and discusses recommendations for using this training modality in the public health workforce.	Mixed methods	Journal article	Information Technology initiative trained professionals in maternal and child health from 13 Southerntier state and local health departments to collect, analyze, and interpret data via a yearlongWeb-based course. The evaluation of this initiative was based on a model of change for health professionals that holds that training influences behavior by increasing knowledge, influencing beliefs related to
Strasser et al.	2013	A Poverty Simulation to Inform Public Health Practice	The purpose of this research was to evaluate the impacts of a poverty simulation for students and practitioners of public health regarding their attitudes toward people living in poverty and awareness of the barriers they face.	Mixed methods	Journal article	A pre- and postsurvey containing Likert scale and qualitative questions was administered to 91 participants.
Matthews & Proctor	2020	Can Public Health Workforce Competency and Capacity be built through an Agent-based Online, Personalized Intelligent Tutoring System?	This research investigated the capability of an agent-based, online personalized (AOP) intelligent tutoring system (ITS) that adaptively uses aptitude treatment interaction (ATI) to deliver public health workforce training in a prescribed health regime and assure their competency.	Mixed methods	Journal article	Potential of an AOP ITS with ATI might have toward supporting surging up the epidemiology and surveillance skill capacity of the public health professional workforce. Use frameworks to understand perception of usefulness of AOP ITS with ATI by PH professionals.
Frahm et al.	2013	Florida Public Health Training Center: Evidence- Based Online Mentor Program	This article presents the fi ndings from an ongoing program designed to evaluate the effectiveness of an online mentoring program to prepare participants for careers in the public health workforce and to develop and maintain FDOH employee professional skills and abilities.	Qualitative	Journal article	The Florida Public Health Training Center (FPHTC) at the University of South Florida (USF) College of Public Health (COPH) developed and implemented an Online Mentor Program (OMP) designed to support and facilitate mentorships among and between Florida Department of Health (FDOH) employees and USF College of Public Health students using a Web-based portal.

Gladman & Perkins	2012	Training Australian general practitioners in rural public health: Impact, desirability and adaptability of hybrid problem-based learning	This study explored the suitability of teaching complex public health issues related to Aboriginal health by way of a hybrid problem-based learning (PBL) model within an intensive training retreat for GP registrars, when numerous trainees have no PBL experience.	Mixed methods	Journal article	A mixed method evaluation. Pre-training surveys on PBL experience and post-training semistructured telephone interviews exploring the impact of the model and its desirability.
Sendall & Domocol	2012	Journalling and public health education: thinking about reflecting...	The purpose of this research is to understand reflective journalling in a first year Public Health practice unit.	Qualitative	Journal article	This research uses pure phenomenography to interpret students' descriptions of reflective journalling.
Robinson & Brownette	2018	Educating public health champions	This article describes a university course that aimed to create public health champions and its evaluation.	Mixed methods	Journal article	The course evaluation aimed to provide a longitudinal understanding of the participants' learning and the impact of the course in terms of developing the
Demers et al.	2011	Creating Opportunities for Training California's Public Health Workforce	To develop a statewide university/community collaborative model for delivering continuing education program.	Quantitative	Journal article	A needs assessment of California's public health workforce was conducted to identify areas of interest, and two continuing education trainings were developed and implemented using innovative distance
Cross et al.	2006	Development of the Public Health Nursing Competency Instrument	This paper describes the development and initial testing of an instrument to measure population-based public health nursing competencies.	Mixed methods	Journal article	The public health nursing competency instrument, consisting of 195 measurable activities organized in the framework of the nursing process, was developed.
Riely et al.	2009	Introducing Quality Improvement Methods into Local Public Health Departments: Structured Evaluation of a Statewide Pilot Project	To test the feasibility and assess the preliminary impact of a unique statewide quality improvement (QI) training program designed for public health departments.	Quantitative	Journal article	A structured distance educationQI training programwas designed and deployed in a first large-scale pilot. To evaluate the preliminary impact of the program, a mixed-method evaluation design was used.
Baker et al.	2009	Examining the Role of Training in Evidence-Based Public Health: A Qualitative Study	(a) to understand the effects and utilization of the material from our EBPH course and (b) to describe more general issues faced by practitioners when using EBPH	Qualitative	Journal article	This article reports on qualitative interviews conducted to evaluate the process and impact of an EBPH course.
Phelps & Johnson	2004	Developing Local Public Health Capacity in Cultural Competency: A Case Study With Haitians in a Rural Community	This project sought to develop a strategy for preparing the public health workforce in a rural Delaware county to provide culturally competent care to an increasing population of Haitians.	Qualitative	Journal article	health department in Delaware created a Web site using Purnell's model for cultural competence (Purnell & Paulanka, 2003) as the organizational framework and rose to the challenge of assisting public health staff to provide culturally sensitive health services to a rapidly increasing population of Haitian immigrants.
Hawley et al.	2007	Academic–Practice Partnerships for Community Health Workforce Development	The WALD Center's partnership and programs provide applied examples for successful academic–practice partnerships for community health practice-based research in the area of workforce development.	Mixed methods	Journal article	The WALD Center's methods provide a model for academic–practice partnerships for community health practice and workforce development, even in environments with scarce health resources.
Jacob et al.	2021	Long-Term Evaluation of a Course on Evidence-Based Public Health in the U.S. and Europe	This study pooled follow-up efforts (5 surveys, with 723 course participants, 2005–2019) to explore the benefits, application, and barriers to applying the evidence-based public health course content.	Quantitative	Journal article	Data were pooled from 5 different surveys that collected similar information from participants of the EBPH course over a 15-year span.

Dean et al.	2014	A Strategic Approach to Public Health Workforce Development and Capacity Building	This paper describes selected WDCB programs implemented by NCHHSTP during the last 4 years in the three strategic goal areas.	Mixed methods	Journal article	Ambassador Program for new hires, career development training for all staff, leadership and coaching for mid-level managers, and a Laboratory Workforce Development Initiative for laboratory scientists. Additionally, the paper discusses three overarching areas—employee communication, evaluation and continuous review to guide program development, and the implementation of key
Chen-Edinboro et al.	2019	Applied Learning for Undergraduates: Integrating NCHEC Competencies and CEPH Public Health Domains in a Public Health Practice Course	The purpose of this manuscript is to describe the development and implementation of this new course.	Mixed methods	Journal article	health program, faculty at the University of North Carolina Wilmington conducted a matrix exercise to assess curricular alignment with the CEPH Domains of Public Health (PHDs) and the National Commission for Health Education Credentialing (NCHEC) competencies.
Meyerson et al.	2018	Learning in the zone: toward workforce development of evidence-based public policy communication	The study objective was to characterize capacity to develop and use EBPC and identify cooperative learning and development opportunities using the case of Human papillomavirus (HPV).	Qualitative	Journal article	informed guided interviews with 27 advocates in Indiana from government, industry, research, state associations and individuals. Participants focused on HPV, cancer, women's health, school health and minority health.
Sridharan et al.	2018	The potential of an online educational platform to contribute to achieving sustainable development goals: a mixed-methods evaluation of the Peoples-uni online platform	This paper reports on an online platform, People's Open Access Education Initiative (Peoples-uni), as a means of enhancing access to master's level public health education for health professionals.	Mixed methods	Journal article	A mixed methods evaluation consisted of two parts, namely an online survey and a telephone interview.
Sudip Bhandari1* , Brian Wahl1, Sara Bennett1, Cyrus Y. Engineer1, Pooja Pandey2 and David H. Peters1	2020	Identifying core competencies for practicing public health professionals: results from a Delphi exercise in Uttar Pradesh, India	This study aims to identify the requisite core competencies for practicing health professionals in mid-level supervisory and program management roles to effectively perform their public health responsibilities in the resource-poor setting of Uttar Pradesh (UP), India	Mixed methods	Journal article	Conducted a multi-step Delphi technique, including a narrative review of competency frameworks, key informant interviews, and workshops to develop competency statements.
Miller, Rambeck & Snyder	2014	Improving Emergency Preparedness System Readiness through Simulation and Interprofessional Education	The primary purpose was to assess the efficiency and effectiveness of this particular educational intervention using best practices in immersive simulation, both the curriculum and the research design were intended to address limitations documented in the research literature.	Mixed methods	Journal article	We collected both quantitative and qualitative data about individual and team knowledge, skills, and attitudes. Content experts designed and pilot-tested scaled quantitative tools.
Fifolt et al.	2022	Reconsidering the "Place" in Field Placement	We describe a specific type of practice experience in which students receive a living allowance, earn academic credit, work onsite, and complete work assignments for their sponsoring organization.	Qualitative	Journal article	In this article, we describe student experiences with remote and hybrid work arrangements, highlight issues regarding equity and inclusion, and discuss implications for future public health practice.
Hoffman & Silverberg	2015	Training the next generation of global health advocates through experiential education: A mixed-methods case study evaluation	This case study evaluates a global health education experience aimed at training the next generation of global health advocates. Demand and interest in global health among Canadian students is well documented, despite the difficulty in integrating meaningful experiences into curricula.	Mixed methods	Journal article	A quantitative survey and an analysis of social network dynamics were conducted, along with a qualitative analysis of written work and course evaluations.

Coombe et al.	2020	Enhancing Capacity of Community–Academic Partnerships to Achieve Health Equity: Results From the CBPR Partnership Academy	To address a critical gap, the Detroit Community-Academic Research Centre designed and implemented the CBPR Partnership Academy, an integrated, yearlong program to enhance capacity of academic–community pairs new to CBPR.	Mixed methods	Journal article	We describe program development, implementation, and evaluation, and analyze results from the first two cohorts.
Miner et al.	2005	The MACH Model: From Competencies to Instruction and Performance of the Public Health Workforce	The Georgia Training Resource and Inventory Network (G-TRAIN) is an example of how the components represented in the MACH Model were used to develop a needs assessment and course management system for Georgia's public health workforce.	Qualitative	Journal article	In 2003, Emory's CPHP partnered with the Georgia Division of Public Health to develop G-TRAIN, a web-based needs assessment and course management system for use in training public health workers. The G-TRAIN system collects needs assessment data and maintains a course catalog of training resources.
Shickle, Stroud, Day, & Smith	2018	The applicability of the UK Public Health Skills and Knowledge Framework to the practitioner workforce: lessons for competency framework development	The initial objective of this study was to develop a public health practitioner apprentice training curriculum.another objective of the study was to assess the extent to which practitioners utilize the competencies defined within the PHSKF. A further objective was to provide guidance on PHSKF competencies not adequately addressed by existing formal (degree course) and informal (on-the-job) training. The final objective was to evaluate the utility of the PHSKF itself.	Qualitative	Journal article	15 small group interviews involving 51 participants were conducted across the 8 health authorities. The interviews also covered appropriateness of the PHSKF as a basis for degree curricula and apprenticeship schemes.Interviews were recorded and transcribed. A deductive approach was used within a framework analysis. Codes were manually applied to these data and combined into themes as appropriate. Interviewees commented on the completed analysis.
Kenefick et al.	2014	On Your Time: Online Training for the Public Health Workforce	The authors describe the On Your Time training series, an effective distance education program and training model for public health practitioners, which includes a standardized process for development, review, evaluation, and continuous quality improvement.	Qualitative	Journal article	The replicable model incorporates what is known about best practices for online training and maximizes available resources in the interests of sustainability.
Hofer et al.	2014	Prevocational exposure to public health in the Kimberley: A pathway to rural, remote and public health practice	To evaluate the Kimberley Population Health Unit (KPHU) prevocational public health placement in terms of its contribution to resident medical officers' (RMOs') knowledge, skills, career path and aspirations.	Quantitative	Journal article	All RMOs who had completed a public health placement at the KPHU (n = 27) during 2001–2012 were invited to complete an online survey in September 2012.
Hamelin & Paradis	2018	Population health intervention research training: the value of public health internships and mentorship	In this paper, we describe the role of internship placements and mentorship for trainees' skills development in population health intervention research and the benefits of embedding research trainees within public health organizations.	Qualitative	Journal article	Two types of interviews were conducted: telephone semi-structured interviews by an external evaluator and face-to-face trainee "exit" interviews by the Program co-director. Semi-annual evaluation reports from each trainee were also reviewed. Qualitative data were subjected to a thematic analysis.
Werner et al.	2005	An Innovation in Partnership Among First Responders and Public Health: Bridging the Gap	This article provides a review of an approach the Heartland Center for Public Health Preparedness took to foster these partnerships and increase the provision of competency-based, integrated responder education and training in the St. Louis, MO, metropolitan area.	Mixed methods	Journal article	To fulfill a mandate to develop an all-hazards disaster response plan, a diverse group of responders came together in a partnership. The partners developed an innovative, competency-based program to reach the many disciplines responsible for emergency management.

Calhoun et al.	2011	Current State in U.S. Public Health Competency-Based Graduate Education	Understanding competency-based education for enhancing educational practices across public health graduate education .	Qualitative	Journal article	have led to the consideration and development of strategies for promoting outcomes-based educational performance and accountability across post-secondary higher education in the US; evolving CBE pedagogy in public health graduate education; and experiences to date regarding success factors, barriers, and challenges encountered with the implementation of
McCullagh	2011	The invisible man e Development of a national men's health training programme for public health practitioners: Challenges and successes	To develop a national men's health training programme for healthcare and social care practitioners to address gender health inequalities and facilitate the effective delivery of health services to men.	Mixed methods	Journal article	A post-training questionnaire was completed by all participants to evaluate the efficacy of the training, levels of satisfaction and impact on future work practice.
Hyder	2020	Teaching systems science to public health professionals	The objective of this study is to increase awareness of systems thinking and systems modeling in public health research and practice.	Mixed methods	Journal article	A short course was offered to public health professionals using a combination of teaching modalities: didactic lectures, group discussions, hands-on programming, and experiential learning.
Galvin et al.	2022	Transnational educational partnerships: achieving public health impact through cross-cultural pedagogical approaches in Haiti	In order to address the increasing need for local public health training and expertise in Haiti, Washington University in St. Louis (WUSTL) partnered with the Université Publique du Nord au Cap-Haitïen, to launch the first undergraduate public health degree program in January 2017.	Mixed methods	Journal article	The development of the curriculum is a result of collaboration between UPNCH and WUSTL, and aims to highlight the importance of transdisciplinary practice, an essential element in confronting complex public health problems of the 21st century.
Asgary	2015	Graduate public health training in healthcare of refugee asylum seekers and clinical human rights: evaluation of an innovative curriculum	An innovative curriculum was developed to equip public health students with appropriate attitude and skills to address healthcare of asylum seekers.	Mixed methods	Journal article	Complementary mixed methods evaluations included pre- and post-curriculum questionnaires, formal curriculum evaluations, final papers and oral presentations
Traynor et al.	2014	Knowledge brokering in public health: a tale of two studies	In this paper, the findings from two studies investigating a Knowledge Broker intervention as a means of enhancing capacity for evidence-informed decision making are presented.	Mixed methods	Journal article	This paper describes work done through a single mixed-methods study (randomized controlled trial with a qualitative component) and a case study.
Bornioli et al.	2020	Evaluation of the UK Public Health Skills and Knowledge Framework (PHSKF): implications for international competency frameworks	This study presents the evaluation of the PHSKF 2016 version, with the aim of reflecting on implications for international public health competency frameworks.	Mixed methods	Journal article	An online survey (n = 298) was completed with stakeholders across the four UK nations and different sectors. This was followed by 18 telephone interviews with stakeholders and survey completers.
Gibbert et al.	2013	Training the Workforce in Evidence-Based Public Health: An Evaluation of Impact Among US and International Practitioners	The objective was to assess use and benefits of the course and identify barriers to using evidence-based public health skills as well as ways to improve the course.	Mixed methods	Journal article	Pre- and post-evaluations among participants who attended from 2008 through 2011 and web-based follow-up surveys among participants who attended from 2005 to 2011.
Mainor et al.	2014	A Systematic Approach to Evaluating Public Health Training: The Obesity Prevention in Public Health Course	This article presents the systematic approach the Center of Excellence for Training and Research Translation (Center TRT) used both to assess practitioners' competencies to lead public health obesity prevention initiatives and to evaluate its annual, competency-based obesity prevention course.	Quantitative	Journal article	Each year, a longitudinal evaluation based on Kirkpatrick's training evaluation framework was administered to course participants (n = 243) to assess perceptions of the course (daily), changes in self-reported competency (immediately pre- and postcourse), and course impact on practice over time (at 6 months).

Granillo et al.	2010	Utilization of the Native American Talking Circle to Teach Incident Command System to Tribal Community Health Representatives	This paper reports on the use of the Native American Talking Circle format as a culturally appropriate method to teach the Incident Command System (ICS).	Mixed methods	Journal article		Incorporating Incident Command System curriculum into a Talking Circle mode that was culturally appropriate. Included a pre-and post-survey.
Steckler et al.	2011	Can health professionals learn qualitative evaluation methods on the World Wide Web? A case example	The goal was to strengthen the technology and analytic skills of the public health workforce.	Quantitative	Journal article		Describes the evaluation of a six unit module to teach public health professionals about qualitative methods.
Gebbie	2008	Competency-to-Curriculum Toolkit	The Competency-to-Curriculum Toolkit is presented to the public health workforce training and education community as an aid in assuring that the workforce, key to the public health infrastructure, is truly competent to perform essential public health services in all areas of public health practice.	Not applicable (grey literature)		Grey Literature	N/A grey literature
de Beaumont	2021	Adapting and Aligning Public Health Strategic Skills	To address new challenges and changing priorities, the de Beaumont Foundation sought to align the Strategic Skills with widely used public health assessments and competency sets.	Not applicable (grey literature)		Grey Literature	Those working in public health must be highly trained in discipline-specific skills as well as cross-cutting, strategic skills to implement effective and ambitious interventions across diverse communities and sectors.
Hunt	2015	Review of Core Competencies for Public Health: An Aboriginal Public Health Perspective	Seeks to address issues of Aboriginal public health including systemic factors related to colonialism, recognition of Indigenous knowledge, and First Nations, Inuit and Metis health governance.	Not applicable (grey literature)		Grey Literature	Culturally relevant Aboriginal health competency frameworks have been used as a measure of analysis against which to discuss the relevance of the PHAC competency framework for Aboriginal public health.
Chong et al.	2022	Embedding experiential learning in undergraduate health promotion curriculum	The integration of academic learning and practice provides students opportunities to increase their knowledge while developing capabilities.	Mixed methods	Journal article		The project-based curriculum, delivered over 12 weeks, consists of theoretical material which students apply through hands-on project planning, implementation and evaluation; projects are carried out on campus.
Saleh et al.	2004	Evaluating the effectiveness of public health leadership training: The NEPHLI experience	We assessed the effect of public health leadership training on the capacity of public health leaders to perform competencies derived from the list of "Ten Essential Public Health Services" presented in 1994 by the steering committee of the Public Health Functions Project.	Quantitative	Journal article		Graduating scholars of the Northeast Public Health Leadership Institute were surveyed to determine differences in skill level in 15 competency areas before and after training.
Coleman, Hudson, & Maine	2013	Health Literacy Practices and Educational Competencies for Health Professionals: A Consensus Study	This study aimed to identify a set of health literacy educational competencies and target behaviors, or practices, relevant to the training of all health care professionals.	Mixed methods	Journal article		Literature review to identify a list of potential competencies and used a modified Delphi to reach consensus
de Lima et al.	2020	Perception and manifestation of collaborative competencies among undergraduate health students	To analyze the perception and manifestation of collaborative teamwork competencies among undergraduate health students who experienced the curricular internship's integration module from the perspective of interprofessional education.	Qualitative	Journal article		qualitative study, developed with the intervention research strategy. Twenty-eight students from five undergraduate health courses participated. Data were collected in three focus group interviews conducted with the undergraduate students at the end of each semester.

Neubauer et al.	2022	Realizing Promising Educational Practices in Academic Public Health: A Model for the Scholarship of Teaching and Learning	This paper presents a conceptual framework and critical considerations for the scholarship of teaching and learning (SoTL) in academic public health.	Qualitative	Journal article	The paper advocates for SoTL as a form of engaged practice and scholarly inquiry that exists in contextually rich, diverse educational environments that abounds with uncertainty.
Edgar et al.	2015	Results from a national survey of health communication master's degree recipients: An exploration of training, placement, satisfaction, and success	This study reports the results of a national survey that targeted the alumni of 5 institutions that offer a master's degree in health communication.	Quantitative	Journal article	We conducted a survey of individuals from across the country who received a master's degree in health communication to better understand the quality of their training and the characteristics of the roles they play within our field.
Chávez et al.	2006	Teaching public health through a pedagogy of collegiality	Principles of communitybased participatory research highlight the importance of building relationships of mutual accountability and emphasize collegial teaching.	Qualitative	Journal article	We present background and theoretical foundations for a pedagogy of collegiality and describe specific teaching methods, classroom activities, and key assignments organized around 4 essential features.
Edgar & Hyde	2005	An alumni-based evaluation of graduate training in health communication: Results of a survey on careers, salaries, competencies, and emerging trends	The current study presents the results of an evaluation of the Master's Program in Health Communication offered collaboratively by Emerson College and the Tufts University School of Medicine.	Mixed methods	Journal article	To conduct the assessment of the program, the two schools collaborated on the development of an on-line survey for the alumni.
Madhok et al.	2018	Building public health capacity through online global learning	In this paper we describe a model for public health capacity building through online Global Learning.	Qualitative	Journal article	We demonstrate how two programmes, Peoples-uni and NextGenU.org, have implemented this model using a mix of low-cost and free online learning courses, a global community of volunteer tutors,
Park, Harrington, Crosswell, & Parvanta	2021	Competencies for Health Communication Specialists: Survey of Health Communication Educators and Practitioners	This research picked up where previous work left off by developing a systematic, comprehensive list of competencies to be broadly considered for graduate programs offering health communication degrees.	Mixed methods	Journal article	Working group drafted a competency list through literature review and expert feedback. Survey to build consensus.
Hagopian, Spigner, Gorstein, Mercer, Pfeiffer, Frey, Benjamin, & Gloyd	2008	Developing Competencies for a Graduate School Curriculum in International Health	The purpose of this article is to report on how we developed international health competencies to guide our curriculum development.	Mixed methods	Journal article	Literature search to find published competency frameworks. Strategic planning for shared values. Environmental scan of other schools. Draft statements circulated.
